# Characterization of the complete mitochondrial genome of Chinese *Konosirus punctatus* (Clupeiformes, Clupeidae) and phylogenetic studies of Clupeiformes

**DOI:** 10.1080/23802359.2020.1823272

**Published:** 2020-09-18

**Authors:** Kun Zhang, Yifan Liu, Xiaolong Yin, Pengxiang Yuan, Jian Chen, Yuanpei Gao, Hongling Ping, Hua Zhang, Zengliang Miao, Bingjian Liu, Pinglin Cao

**Affiliations:** a Marine Science and Technology College, Zhejiang Ocean University, Zhoushan, China; b Zhoushan Fisheries Research Institute of Zhejiang Province, Zhoushan, China; c Department of Food Science and Pharmacy, Zhejiang Ocean University, Zhoushan, China; d Key Laboratory of Tropical Marine Bio-resources and Ecology, Chinese Academy of Sciences, Beijing, China

**Keywords:** *Konosirus punctatus*, mitochondrial genome, phylogenetic analysis, Characiformes

## Abstract

The Dotted Gizzard Shad (*Konosirus punctatus*) was one of the most important commercial fish species in China, Japan and Korea. In this study, the complete mitochondrial genome of *K. punctatus* was presented. The full length of the mitochondrial genome was 16,705 bp, including 13 protein-coding genes (PCGs), two ribosomal RNAs, 22 transfer RNA genes, one non-coding control region (CR) and one origin of replication on the light-strand. The total nucleotide composition of mitochondrial DNA was 25.79%A, 25.09%T, 29.05%C, 20.08%G, and AT was 50.88%. The mitochondrial genome provides an important resource for solving taxonomic problems and studying molecular evolution.

The Dotted Gizzard Shad (*Konosirus punctatus*) in the family Clupeidae (Clupeiformes) was distributed along all coasts of Korea, China, Japan, and Taiwan (Kong et al. 2004; Myoung and Kim 2014). *K. punctatus* was one of the most important commercial fish species in China (Song et al. 2017), Japan (Kong et al. 2004) and Korea(Myoung and Kim 2014). *K. punctatus* was a small pelagic fish of warm temperate zone in estuaries, generally living in soft sandy silt environment (Jin 1993; Xu et al. 2013). In this study, we described the complete mitochondrial genome of *K. punctatus* and explored the phylogenetic relationship within Clupeiformes, to gain its molecular information and thus contribute to facilitate future studies on population genetic structure and phylogenetic relationships.

In order to obtain wild *K. punctatus*, individuals were collected from the natural sea (Zhoushan, Zhejiang Province, China, N29°32′42.60″, E122°26′54.97″) stored in laboratory of Zhejiang Ocean University with accession number 20190825bjy20. Total genomic DNA was extracted using a phenolchloroform extraction protocol (Sambrook et al. 1982). Subsequently, based on the existing complete mitochondrial gene of *Amblygaster sirm* (AP018760), 21 pairs of primers were designed, the samples were amplified by PCR, and then sequenced using Sanger sequencing technology. The complete mitochondrial genome was annotated using Sequin version 15.10 (http://www.ncbi.nlm.nih.gov/Sequin) and tRNAscan-SE version 2.0 (http://trna.ucsc.edu/tRNAscan-SE/) (Lowe and Eddy 1997). Like typical vertebrate mitochondria (Guo et al. 2017; Zhu et al. 2018), the mitochondrial genome of *K. punctatus* was also a closed double-stranded circular molecule consisting of 16,705 nucleotides (GenBank accession number: MT801134.1), which was within the length of other bony fish mitochondrial genomes (Boore 1999). Compared with the study by Lavoue et al. (Sébastien et al. 2013), we had provided a new and more accurate sequence with a difference of 7 bases. The complete mitochondrial genome contains 13 protein-coding genes (PCGs), two ribosomal RNA genes (12 s and 16 s), 22 transfer RNA (tRNA) genes, a putative control region (CR) and one origin of replication on the light-strand (OL). The overall base composition was A (25.79%), T (25.09%), C (29.05%), G (20.08%), respectively, with a slight AT bias (50.88%). *K. punctatus* mitochondrial genes were mostly encoded on the heavy strand, except for ND6 in 13 PCGs and eight tRNA (Gln, Ala, Asn, Cys, Tyr, Ser^AGT^, Glu, and Pro) genes on the light strand coding. The start codons of the 13 PCGs encoding genes were ATG except for COI which was GTG, which is quite common in vertebrate mtDNA (Wang et al. 2008). The start codons of the 13 PCGs encoding genes are ATG except for COI which is GTG. The genes with TAG as the stop codon were ND1 and ND6, the genes with a single T as the stop codon were ND2, COI, COII, ATP8, ND3, ND4 and Cytb, and the genes with TAA as the stop codon were ATP6, COIII, and ND5. Transfer RNA genes and their potential cloverleaf structures were identified using tRNAscan-SE (Lowe and Eddy 1997). The lengths of 22 tRNAs distributed on the H and L strands were between 66 and 74. Except for tRNA^Ser (GCT)^ lacking a DHU stem among the 22 tRNAs, the remaining tRNAs can form a common clover secondary structure (Sprinzl et al. 1998). The lengths of the two rRNA genes are 952 bp (12srRNA) and 1682 bp (16srRNA) respectively, which located between the tRNA^Phe^ and tRNA^Leu(TAA)^ and separated by the tRNA^Val^ gene. The length of the control region was 1042 bp, located between tRNA^phe^ and tRNA^Pro^.

Based on the 13 protein coding sequences of 38 species' mitochondrial genomes, we used Bayesian inference (BI) and maximum likelihood (ML) to construct a phylogenetic tree. According to the Akaike Information Criteria (AIC), the most suitable nucleotide sequence model was selected through MrModeltest 2.3 (Yamaoka et al. 1978), and finally the most suitable model was GTR + I + G. Two phylogenetic trees were built using software MrBayes (Ronquist et al. 2012) and PhyML (Guindon et al. 2010). The results showed that the two trees were basically identical in topology structure, so only one topology with two supporting values was displayed, including the bootstrap value of ML tree and the posterior probability of Bayesian analysis. Both phylogenetic trees indicate that *K. punctatus* was closely related to *Amblygaster sirm* (Figure 1).

**Figure 1.**
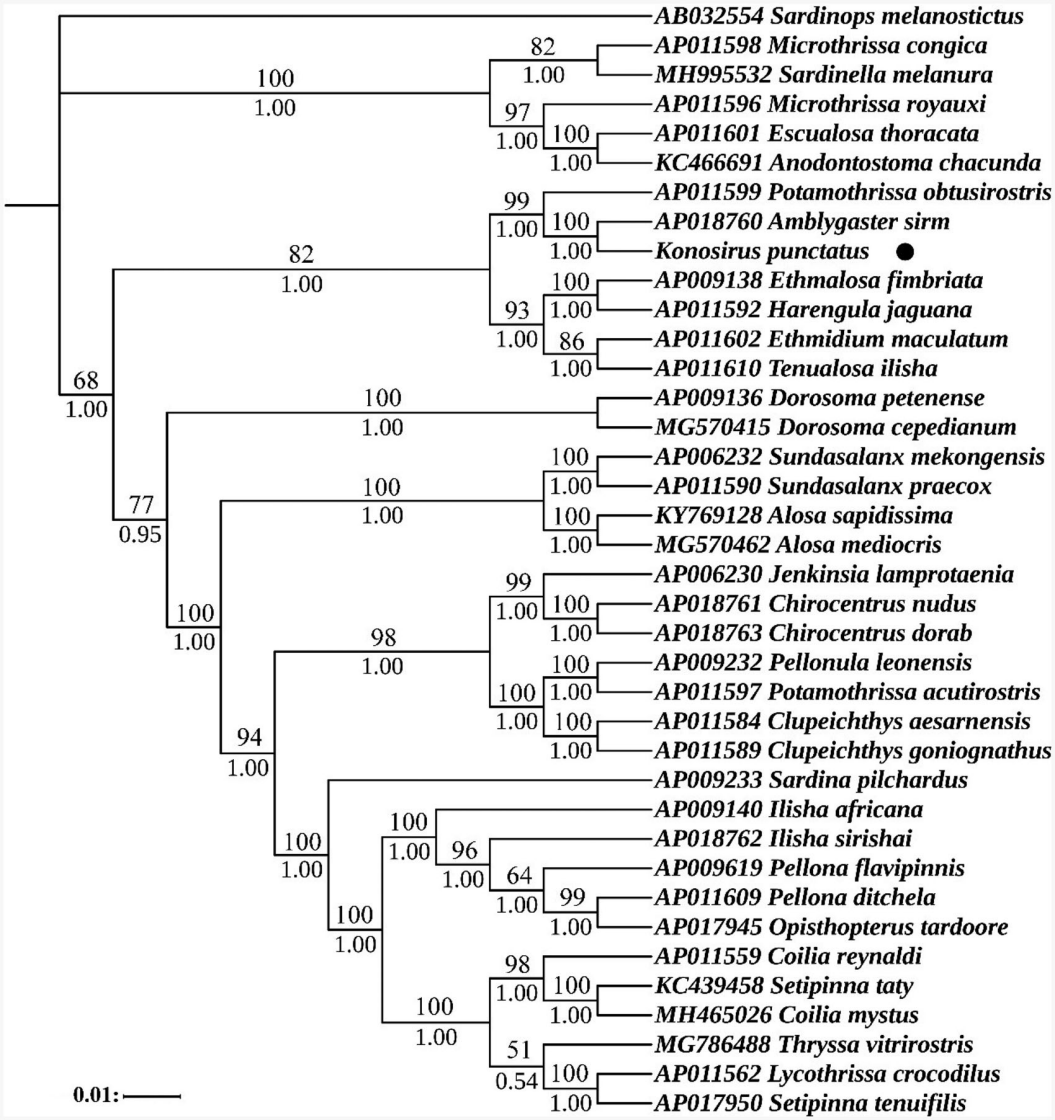
Phylogenetic analysis based on the nucleotide sequences of the 13 PCGs in the mitogenome. Support values for the Bayesian analyses (Bayesian posterior probabilities with 10 million generations; discarding 25% as burnin) and Maximum Likelihood analyses (bootstrap support with 1000 replications) are shown next to nodes. The number before the species name was the GenBank accession number. The numbers beside the nodes are posterior probabilities (BI, bottom) and bootstrap (ML, top). The genome sequence in this study was labeled with a black dot.

## Data Availability

The data that support the findings of this study are openly available in “NCBI” at https://www.ncbi.nlm.nih.gov/nuccore/MT801134.1, GenBank accession number: MT801134.1.
